# Tuberculosis Notes

**Published:** 1919-07-05

**Authors:** 


### TUBERCULOSIS NOTES

### Big Four Wanted.

In the matter—the pressing matter—of provision for tuberculous ex-combatants, who at present are excluding civilian consumptives from the sanatoriums which ought to be receiving them under the National Insurance Act, the President of the Local Government Board and the Minister of Pensions have appointed a committee of fourteen, mainly M.P.s (Major Waldorf Astor, chairman), to consider and report upon immediate practical steps for provision of residential treatment for discharged consumptive soldiers and sailors, and for their reintroduction into employment, especially upon the land. In view of the necessity for speedy action, it would be as well if it were possible to appoint from these fourteen a big four—or even three.

### Chelmsford Anti-Tuberculosis Conference.

A useful campaign with the above object was inaugurated last week by the Essex Insurance Committee at the Shire Hall, Chelmsford. The Mayor presided, and was supported by the Chairman of the local District Council, and by Dr. Bullough, the lately appointed County M.O.H., vice Dr. Thresh, retired. Dr. H. Platts, clinical adviser to the Committee, set forth well-known facts, as to-day accepted, as to the demography of tuberculosis. He was, however, rather too terroristic, as in saying (or at least in being reported, which has the same effect) that 70 per cent. of persons over thirty were tuberculous. The layman, who cannot be expected to know that this refers only to minute healed lesions of no practical import, is apt to be made tuberculo-phobic by statements of this kind. Also it is exaggeration to say that during the war, in Germany "whole towns were practically wiped out by the disease." Has Dr. Platt had first-hand evidence of this? Because the ordinary daily newspapers during the war, as they themselves confess, have been converted into lie-factories; the only true thing in a certain one of them—so runs the *mot*—is its date. But here at hand is a summary, entitled *Die Aushungerung Deutschlands*, of the December discussion on this subject at the Berlin Medical Society. The tuberculosis mortality was twice as great in 1918 as in 1913; also it was of severer form, with an especial frequency of tuberculous meningitis amongst children. But tuberculosis mortality in England has sadly increased too. Witness how packed to overflowing every sanatorium is, while three or four months is a common period to have to wait for admission thereto. Yet no town has been visibly affected here, nor is the epidemiology of the disease such as to make it possible for populations to be "wiped out" by it in two or three years, not even in the case of the non-resistant savage.

### Developments of Lung Collapse Therapy.

In a recent number of the *Tuberkulose-Fürsorge Blätter* Unverricht refers again to the application of the artificial pneumothorax treatment of consumption to the old problem that disease presents when complicated with pregnancy. In the *Deutsche Medzinische Wochenschrift*, 1917, No. 50, he had communicated two instances in which the induction of abortion seemed unavoidable, since pyrexial lung tubercle was present with hæmoptysis. However, it was unilateral, and an artificial pneumothorax produced in both cases stay of the disease, with, in the one instance (at the date of writing) successful delivery. In all probability these two cases are the ones cited in his second paper; the other patient also went to term. It is recommended that such a pneumothorax should be maintained for eighteen months or two years. In view of cases like these, Voornveld claims that when it is unilateral, or fairly unilateral, phthisis that complicates pregnancy, abortion should only be done if artificial pneumothorax is impracticable or fails. More and more do the various specialties tend to meet on their confines.

### A Remote Danger of Thoracocentesis.

A Swiss surgeon describes local tuberculosis of the chest wall developing at the site of a puncture made, in one instance six months, in the other nearly two years, previously, for the evacuation of a pleural effusion. Of course pleural effusions in the great majority of cases imply underlying pulmonary tuberculosis. He cites Henschen's text-book for first mention of this risk: Local tuberculosis of the chest wall has followed even an exploratory puncture. Lucius Spengler, of Davos, commenting upon this paper, notes that the literature of artificial pneumothorax therapy contains many instances of the sequela under notice, for artificial pneumothorax is unfortunately often complicated by pleural effusion. In treating a chronic tuberculous empyema by aspiration, evacuation should be done as completely as possible (the pus being replaced by air) and reaccumulated fluid never allowed to rise to the site of the puncture. This also helps to prevent the formation of fistulæ, internal as well as external.

